# Barriers to and Facilitators of Telemedicine in Rural Healthcare in Japan: A Qualitative Analysis

**DOI:** 10.7759/cureus.110032

**Published:** 2026-06-01

**Authors:** Daisuke Matsubara, Yukiko Honda, Kazuhiko Kotani

**Affiliations:** 1 Department of Community and Family Medicine, Jichi Medical University, Shimotsuke, JPN; 2 Department of General Medicine, Nagasaki University Graduate School of Biomedical Sciences, Nagasaki, JPN; 3 Department of Social Epidemiology, Graduate School of Medicine and Faculty of Medicine, Kyoto University, Kyoto, JPN

**Keywords:** barriers, enabler, facilitators, issues, japan, remote healthcare, teleconsultation, telemedicine

## Abstract

Background

Telemedicine can be used to improve healthcare access. However, it is used less frequently in Japan, particularly in the rural areas. Rural healthcare systems in Japan are unique in that medical services are delivered through a collaboration between core hospitals and rural clinics. Rural clinics provide primary care, whereas core hospitals are expected to provide support through telemedicine-based inter-professional communication. This study qualitatively investigated the major barriers and facilitators of telemedicine adoption in rural settings, particularly from the perspective of core hospitals that support rural clinics.

Methods

One-on-one semi-structured interviews were conducted at nine core hospitals that used or were ready to use telemedicine systems. The interviews investigated barriers to and facilitators of telemedicine adoption.

Results

Three barriers and 10 facilitators were identified regarding the telemedicine adoption. These barriers included: 1) budgetary, 2) human resources, and 3) operational issues. Facilitators were conceptually grouped into three domains: 1) institutional and policy-level support (incentives, establishment of operational rules and guidelines, consultation services by local governments, use of consultants, and preparation of trouble response teams), 2) professional and workforce development (human resource education), and 3) community and cultural acceptance (user-friendly systems, collaboration across communities, overcoming field-level apprehension, and resident comprehension).

Conclusions

While barriers largely reflected structural constraints, facilitators were identified across the institutional, professional, and community levels. Among these, the development of user-friendly systems emerged as a potentially important facilitator in the context of telemedicine adoption in rural Japan. These findings may provide useful insights for promoting telemedicine adoption in core hospitals supporting rural healthcare in Japan.

## Introduction

Telemedicine is defined as the use of information and communication technologies (ICT) in healthcare, and has expanded in recent years [1,2]. Telemedicine is commonly described as services delivered directly to patients (doctor-to-patient), while it may also include inter-professional consultations between healthcare providers (doctor-to-doctor) [2]. Telemedicine enables the delivery of healthcare services across distance and time, and is reported to improve healthcare access, enhance continuity and quality of care, reduce costs, and increase the efficiency of healthcare systems [2-5]. These advantages may be particularly relevant in rural areas where medical resources are limited [6-8].

In the Japanese rural healthcare system, medical services are delivered through collaboration between core hospitals and rural clinics [9]. Core hospitals support rural clinics by providing specialized care and dispatching physicians [9]. Rural clinics provide primary care in isolated areas where no other medical facilities exist within a four-kilometer radius, with a population of 1,000 or more, and where access to the nearest medical institution requires more than 30 mins of travel [9]. As physicians in rural clinics often practice alone and provide comprehensive care across a broad spectrum of conditions, support from core hospitals is essential for maintaining access to specialized care [10]. In this context, telemedicine is expected to play an important role in strengthening inter-institutional collaboration [11]. This structural context in Japan makes telemedicine unique in that it is discussed as a tool not only for direct patient care, as commonly described in many international studies, but also for inter-professional consultations between healthcare institutions.

However, telemedicine adoption in rural settings in Japan remains limited. A nationwide survey of rural clinics in Japan reported that telemedicine adoption was 19% in 2020, with inter-professional consultations being the most common form of communication (50%) [11]. Another nationwide survey conducted in 2022 showed that telemedicine adoption in core hospitals was approximately 25% and was more frequently used for inter-professional communication (85.7%) than direct patient care (28.6%) [12]. These findings suggest that telemedicine in core hospitals may function as a tool for inter-professional communication to support rural clinics.

International comparisons have highlighted differences in telemedicine adoption patterns across healthcare systems. In the United States, telemedicine adoption for direct patient care was 29.7% in rural areas compared to 40.2% in large central metropolitan areas (p<0.0010) in 2022 [13]. A national study in Australia identified key barriers to telemedicine adoption including funding, time, infrastructure, equipment, skills, and preferences for traditional approaches [14]. Previous reviews on telemedicine from the United States and international settings have highlighted concerns regarding data accuracy, patient privacy, and the cost- and time-saving benefits of remote consultations in rural areas [15,16]. We previously reported that collaboration with local governments could facilitate telemedicine adoption in the rural areas of Japan [12]. However, because healthcare systems differ between countries, context-specific factors influencing telemedicine adoption in Japan remain poorly understood, particularly from the perspective of core hospitals supporting rural clinics through inter-professional communication [17,18].

A qualitative approach is considered appropriate for exploring the context-specific barriers and facilitators of telemedicine adoption that could not be fully captured through quantitative surveys [19]. Therefore, we aimed to qualitatively investigate organizational, operational, and community-related barriers to and facilitators of telemedicine adoption among institutional personnel in core hospitals supporting rural healthcare, using a thematic analysis of semi-structured interviews. This study may provide useful insights for promoting telemedicine in rural healthcare in Japan.

## Materials and methods

Rural healthcare in this study referred to healthcare delivery in geographically remote areas in Japan, where core hospitals support rural clinics with limited access to specialized healthcare resources [9]. This qualitative study was conducted in nine rural core hospitals in Japan. Rural medicine experts purposively identified core hospitals that had previously participated in rural healthcare research and were considered capable of providing information-rich perspectives regarding telemedicine implementation. Hospitals were eligible if they had either implemented telemedicine systems (n=3) or were preparing to implement them in routine practice (n=6), with operational preparation largely completed in the latter institutions. Participating hospitals were located in geographically diverse rural regions across Japan. In March 2023, one-on-one semi-structured interviews were conducted with institutional personnel who were most knowledgeable about telemedicine. The interviews explored telemedicine implementation, including perceived barriers, facilitators, and related needs. Each interview lasted approximately 60 mins. This study was approved by the Jichi Medical University Bioethics Committee for Medical Research (No. 22-157). Written informed consent was obtained from all participants.

All interviews were audio recorded and transcribed verbatim. Inductive semantic thematic analysis was conducted following the six-phase method described by Braun and Clarke in 2006 [19]: 1) familiarization with the data, 2) generation of initial codes, 3) searching for themes, 4) reviewing themes, 5) defining and naming themes, and 6) producing reports. Initial coding and theme development were primarily conducted by one author (YH), who was involved in the interviews and data familiarization process. The identified themes and interpretations were subsequently discussed among the authors to enhance interpretive validity and investigator triangulation. Experts in rural medicine contributed to the contextual interpretation of the data, and efforts were made to minimize interpretive bias through discussions among the authors. MAXQDA 2020 Analytics Pro (VERBI Software, Berlin, Germany) was used for data analysis.

In this study, telemedicine was defined as online patient information sharing and inter-professional communication (doctor-to-doctor), and direct patient care (doctor-to-patient with nurses) based on the national classification framework in Japan [20].

## Results

Participants included physicians (n=5) and administrative staff (n=4) (Table 1).

**Table 1:** Details of the participants

Institutional personnel	Category	N	%
Sex	Male	9	100
Age	30s	1	11.1
	40s	1	11.1
	50s	2	22.2
	60s	4	44.4
	Unknown	1	11.1
Profession	Physician	5	55.6
	Administrative staff	4	44.4
Service in a hospital (years)	0-3	2	22.2
	4-7	2	22.2
	8-26	2	22.2
	27 or more	3	33.3

Three major barriers and 10 facilitators regarding telemedicine adoption were identified (Table 2).

**Table 2:** Barriers to telemedicine adoption

Themes	Representative quotes
Budgetary issues	“We received a grant from the local government when we established the system, but it will not be renewed. We will have to fund it with our own budget next year.” (physician)
	“We can budget for it, but maintenance is a challenge. The annual maintenance costs are quite expensive, and in some cases the system has been stopped or reconsidered.” (administrative staff)
Human resource issues	“It is important build a system after me. I have to teach that know-how to someone else, and that is difficult.” (physician)
	“Healthcare resources are decreasing nationwide, and rural areas cannot compete with urban areas in this national competition for talent.” (physician)
	“There are not enough home care nurses, and we are unable to meet the demand.” (administrative staff)
Operational issues	“It would be helpful to have a national organization or group of people that can provide support and advice and is easy to rely on.” (physician)
	“Although time was allocated to replace the hardware, considering the low frequency of use, we discussed whether it was worth maintaining.” (administrative staff)

These barriers included: 1) budgetary issues, 2) human resources, and 3) operational issues. The 10 facilitators were conceptually grouped into three domains: 1) institutional and policy-level support, 2) professional and workforce development, and 3) community and cultural acceptance (Table 3).

**Table 3:** Facilitators of telemedicine adoption ICT: Information and Communication Technology.

Domain	Theme	Representative quotes
Institutional and policy-level support	Incentives	“If additional reimbursement were provided for employing ICT technicians, it would make it easier to introduce ICT and encourage people to move in that direction.” (administrative staff)
		“If young physicians were appropriately compensated for after-hours work related to network coordination or telemedicine system management during their clinical duties, they would be more willing to engage in such activities.” (physician)
	Establishment of operational rules and guidelines	“Depending on the size of the hospital, it might be helpful to have guidance regarding the appropriate levels of system collaboration and workload required for institutions of different sizes.” (physician)
		“Regarding telemedicine, the guidelines and regulations are constantly changing. Keeping up with them alone requires considerable effort.” (physician)
	Consultation services by local governments	“There is a contact point in the city where you can seek advice. We consult with them about how to utilize various subsidies.” (administrative staff)
		“There is a division in XX City called the Medical Division. Representatives from each hospital visited there and discussed various issues with them.” (administrative staff)
	Use of consultants	“Without consultants, we wouldn't have been able to implement the system on our own.” (physician)
		“The consultants managed the project for us, which was extremely helpful.” (physician)
	Preparation of trouble response teams	“We don't want to be in a situation where we are unable to do anything the moment the computer system stops working.” (physician)
		“We have manuals for responding to computer system shutdowns; therefore, we can immediately switch to paper charts and paper-based test orders when necessary.” (physician)
Professional and workforce development	Human resource education	“We want to hire personnel who are knowledgeable about both information technology and medicine.” (administrative staff)
		“It would be ideal to have personnel who can handle both clinical work and information management.” (physician)
		“Medical informatics education should become a standard component of medical school education.” (physician)
Community and cultural acceptance	Development of user-friendly systems	“If frontline staff do not find the system easy to use, it will not be effective in practice.” (administrative staff)
		“It would be helpful to have systems that are easy to use, even for people who are unfamiliar with electronic devices, provided they receive a small amount of guidance.” (physician)
	Collaboration across communities	“I think the most important thing is how to share issues with everyone.” (physician)
		“I think the medical, dental and pharmacy associations must collaborate, along with the fire department, the police, and local governments.” (physician)
	Overcoming field-level apprehension	“There is some resistance to changing existing practices and introducing new systems; therefore, we need to build confidence and ensure that the entire hospital understands that the system can be successfully used.” (administrative staff)
		“We are conscious of reducing the sense of difficulty experienced in the field. Without that, no matter how useful or convenient the system may be, it will not function effectively.” (administrative staff)
	Resident comprehension	“The sustainability of local healthcare is difficult without the understanding and support of local residents.” (administrative staff)
		“We need to inform local residents about telemedicine. Recently, some patients who were already aware of such services have begun asking about them.” (physician)

Barriers to telemedicine adoption

Three major barriers were identified: budgetary, human resource, and operational barriers (Table 2).

Budgetary Issues

Although the initial adoption costs are often supported by public subsidies, operational and maintenance expenses tend to be the responsibilities of individual institutions. As one physician noted, “We received a grant from the local government when we established the system, but it was not renewed. We will have to fund it with our own budget next year.” Several participants expressed concerns regarding the financial burden of ongoing system updates.

Human Resource Issues

The participants highlighted their reliance on key personnel and the difficulty of succession planning. As one physician stated, “I have to teach that know-how to someone else, and that is difficult.” The shortage of physicians and nurses further limits the feasibility of telemedicine operations, particularly in rural areas that compete with urban centers for staff.

Operational Issues

Not only technical maintenance burdens but also difficulties in sustaining routine telemedicine operations because of the low frequency of use in rural settings were reported. As one administrative staff mentioned, “Considering the low frequency of use, we discussed whether it was worth maintaining.” Participants also expressed a need for centralized support systems.

Facilitators of telemedicine adoption

The 10 facilitators were conceptually grouped into three domains (Table 3).

Institutional and Policy-level Support

This domain includes incentives, the establishment of operational rules and guidelines, consultation services by local government, the use of consultants, and the preparation of trouble response teams. Participants emphasized that institutional incentives and external support were important for introducing and sustaining telemedicine systems. As one administrative staff member noted, “If additional reimbursements were provided for employing ICT technicians, it would make it easier to introduce ICT and encourage people to move in that direction.” Participants also emphasized the importance of institutional consultation and support systems. One administrative staff explained, “There is a contact point in the city where you can seek advice. We consult with them about how to utilize various subsidies.” These structural mechanisms were considered important for sustaining telemedicine implementation.

Professional and Workforce Development

This domain includes human-resource education. The shortage of personnel capable of bridging clinical practice and information technology was highlighted repeatedly. One physician commented, “It would be ideal to have personnel who can handle both clinical work and information management.” Several participants suggested that national- or university-level educational frameworks should incorporate medical informatics training to strengthen workforce capabilities.

Community and Cultural Acceptance

This domain includes the development of user-friendly systems, collaboration across communities, overcoming field-level apprehension, and improving resident comprehension. Participants emphasized the importance of developing universally accessible systems that are easy for frontline staff and patients. As one administrative staff mentioned, “If frontline staff do not find the system easy to use, it will not be effective in practice.” Participants also described resistance to changing existing practices and emphasized the importance of building confidence in telemedicine use within communities. One administrative staff noted, “There is some resistance to changing existing practices and introducing new systems; therefore, we need to build confidence and ensure that the entire hospital understands that the system can be successfully used.” In addition, collaboration across communities and organizations, including medical associations, emergency services, and local governments, was considered important for sustaining regional telemedicine systems. Understanding and support from local residents were also viewed as essential for sustaining telemedicine and local healthcare systems in rural communities.

## Discussion

This qualitative study identified three major barriers - budgetary, human resources, and operational issues - and 10 facilitators across three domains: institutional and policy-level support, professional and workforce development, and community and cultural acceptance. Barriers reflected structural constraints, whereas facilitators extended across multiple layers of the healthcare system, including the institutional, professional, and community levels.

The Cochrane framework provides a map to help interpret these findings [21,22] (Table 4). 

**Table 4:** Mapping of the identified barriers and facilitators onto the Cochrane domains ● indicates conceptual correspondence between each category in the current study and the respective Cochrane domain. The mapping was based on [22].

Themes	Domain	Category	Cochrane domains					
			Cognitive/behavioral domain	Attitudinal/rationale-emotive domain	Healthcare professional/physician domain	Clinical practice guidelines/evidence domain	Patient domain	Support/resource domain
Barriers		Budgetary issues						●
		Human resource issues						●
		Operational issues		●		●		●
Facilitators	Domain 1: Institutional and Policy-Level Support	Incentives	●					●
		Establishment of operational rules and guidelines				●		
		Consultation services by local government						●
		Use of consultants			●			●
		Preparation of trouble response teams	●					●
	Domain 2: Professional and Workforce Development	Human resource education	●		●			●
	Domain 3: Community and Cultural Acceptance	Collaboration across communities					●	●
		Overcoming field-level apprehension		●				
		Resident comprehension						●
		User-friendly systems	Not applicable					

The mapping to the Cochrane domains was performed after the inductive thematic analysis to facilitate interpretation of the identified themes, rather than as a primary analytic framework. As this mapping was interpretive, several themes could plausibly relate to multiple Cochrane domains depending on the implementation context. The three barriers identified were primarily concentrated within the support/resource domain, reflecting structural constraints. In contrast, the facilitators were distributed across all six Cochrane domains: cognitive/behavioral, attitudinal/rationale-emotive, healthcare professional, clinical practice guideline/evidence, patient, and support/resource, suggesting that successful telemedicine adoption may require involvement across multiple layers of the healthcare system. In particular, the development of user-friendly systems did not fit clearly within a single Cochrane domain (Table 4), as its meaning and relevance appeared to vary according to the context of implementation, including clinical workflow, patient accessibility, and technological usability. 

The most frequent barriers to telemedicine adoption, identified in our previous quantitative survey in Japan [12], were hardware preparation and financial issues, followed by securing human resources, which aligns with the results of this study. Although telemedicine adoption can be supported by local government [12], the current qualitative study further clarified that financial barriers might extend beyond the initial costs to ongoing maintenance and system renewal, highlighting the potential need for long-lasting local governmental support. In addition, the Japanese health insurance system provides limited reimbursement for ICT infrastructure in core hospitals, even though telemedicine, in the form of inter-professional communication, is expected to support rural clinics within the national rural healthcare framework [23]. This mismatch between institutional expectations and financial support may have contributed to the financial barriers observed in this study. Barriers to human resources included workforce shortages and dependence on key personnel with multiple skills bridging healthcare and ICT, which may be particularly prominent in rural settings where medical professionals are limited [6-8]. Operational barriers included a lack of support systems and low frequency of telemedicine use, which were reported to be less prominent in a previous quantitative study [12]. This suggests that operational issues may be under-recognized in traditional quantitative research. Similarly, structural issues have been reported internationally [14], indicating that the Japanese context shares common challenges with other countries, regardless of differences in healthcare systems or socioeconomic backgrounds. As these barriers largely reflected structural constraints, sustained policy support may be important for telemedicine adoption in rural settings, rather than relying on individual institutional efforts.

In contrast to the relatively concentrated barriers, facilitators were widely distributed across the institutional, professional, and community levels. At the institutional level, collaboration between local governments [12] and reimbursement policies may promote telemedicine adoption. At the professional level, education and cultivation of multi-skilled personnel bridging clinical practice and ICT could be considered important policy approaches, potentially reducing reliance on individual key persons [6]. In addition, such educational support may help facilitate inter-professional communication by enabling physicians in rural clinics, who often practice alone, to access specialist consultation and clinical guidance through ICT-based communication with core hospitals [9,10,23]. Community acceptance was perceived as an important facilitator of telemedicine adoption in rural settings. Collaboration across communities, overcoming field-level apprehension, and residents’ comprehension were considered important factors. These findings suggest that telemedicine adoption in rural settings is not purely a technical or organized process but may also involve socially negotiated processes within local communities. Even when structural and professional frameworks were well developed, participants perceived that telemedicine implementation could remain challenging without understanding and collaborating with local residents, consistent with the broader theory of healthcare implementation [24].

Notably, the present findings should be interpreted in the unique context of the functional relationship between core hospitals and rural clinics in Japan, where telemedicine adoption in core hospitals is intended to support rural clinics through inter-professional communication [9,23]. This characteristic may explain why the identified barriers were largely institutional rather than primarily related to patients. In addition, as previous studies on telemedicine have predominantly focused on direct patient care, evidence regarding inter-professional telemedicine remains limited. In this context, a review paper focusing on inter-professional consultations reported that infrastructure-related factors were most frequently identified, while patient-related factors were less commonly addressed, which aligns with the results of the present study [2]. Furthermore, given that Japan’s healthcare system is closely linked to local governments, hospitals, and communities, inclusive processes may be important for telemedicine adoption in rural settings. Rural areas in Japan are facing rapid population aging and are characterized by variations in digital literacy [25]. Internet use among individuals aged 13-59 exceeded 90% in 2020, whereas it was low among 59.6% of those aged 70-79 and 25.6% of those aged 80 years or older [25]. In addition to infrastructure-related factors discussed above, the usability of telemedicine systems may also play an important role in inter-professional consultations [2]. This context may partly explain why the present study highlighted the practical importance of user-friendly systems in inter-professional telemedicine adoption in rural Japan, particularly in settings where core hospitals support rural clinics and digital literacy among older residents may be variable. As the Cochrane domains represent an internationally recognized framework for implementation, further research is required to clarify how user-friendly systems should be positioned within telemedicine implementation frameworks in rural settings in Japan.

The current study had several limitations. First, the relatively small number of participants may limit the generalizability of the findings and complete thematic saturation cannot be definitively confirmed. However, semi-structured interviews were conducted, and the consistency of the themes suggested that the major findings reflected common barriers and facilitators relevant to telemedicine adoption. Second, although participating institutions were at different stages of telemedicine implementation and included both physicians and administrative staff, no major differences were observed in the identified themes, according to implementation stage or professional background. In addition, telemedicine in this study included multiple modalities, such as inter-professional communication, online patient information sharing, and doctor-to-patient care with nurses. Barriers and facilitators may differ according to specific telemedicine modalities, clinical workflows, and use cases. Third, participants consisted of male institutional representatives most knowledgeable about telemedicine implementation at each core hospital. Therefore, the findings primarily reflect institutional perspectives from core hospitals, and may not fully represent those of other stakeholders involved in telemedicine adoption, such as nurses, patients, residents, local government officers, information technology staff, or physicians working in rural clinics. In addition, because participating institutions had previous involvement in rural healthcare research and were considered capable of providing information-rich perspectives regarding telemedicine, selection bias toward institutions with greater interest in telemedicine may have been present. Furthermore, socially desirable responses regarding telemedicine adoption may have influenced the findings. Finally, because this study was conducted within the unique rural healthcare system in Japan, and regional differences in healthcare infrastructure and delivery systems may exist, caution is required when transferring these findings to other healthcare systems or countries. In addition, the authors’ professional backgrounds in rural medicine and telemedicine research may have influenced interpretation of the findings. 

## Conclusions

This qualitative study identified barriers to and facilitators of telemedicine adoption in core hospitals supporting rural clinics in Japan. While barriers largely reflected structural constraints, facilitators were identified across the institutional, professional, and community levels. Among these, the development of user-friendly systems emerged as a potentially important facilitator in the context of telemedicine adoption in rural Japan. These findings may provide useful insights for promoting telemedicine adoption in core hospitals supporting rural healthcare in Japan. Future studies involving multiple stakeholders across rural healthcare settings are needed to further clarify barriers to and facilitators of telemedicine implementation and long-term sustainability.
